# Associations of menopausal age with virological outcomes and engagement in care among women living with HIV in the UK

**DOI:** 10.1080/25787489.2020.1852817

**Published:** 2020-12-08

**Authors:** Hajra Okhai, Shema Tariq, Fiona Burns, Yvonne Gilleece, Rageshri Dhairyawan, Teresa Hill, Caroline A. Sabin

**Affiliations:** 1 Institute for Global Health, University College London, London, UK; 2 National Institute for Health Research (NIHR), Health Protection Research Unit (HPRU) in Blood-borne and Sexually Transmitted Infections, University College London, UK;; 3 Royal Free London NHS Foundation Trust, London, UK; 4 Brighton & Sussex University Hospitals NHS Trust, Brighton, UK; 5 Brighton & Sussex Medical School, Brighton, UK; 6 Department of Infection and Immunity, Barts Health NHS Trust, London, UK

**Keywords:** HIV, ageing, women, menopause, antiretroviral therapy, viral outcomes, viral suppression, viral rebound, engagement in care

## Abstract

**Background:** Women ageing with HIV undergo sex-specific changes. There is limited evidence available with regards to how the menopause impacts HIV outcomes.

**Objective:** To investigate whether menopausal age is associated with engagement-in-care (EIC), viral load (VL) suppression and rebound among women living with HIV.

**Methods:** Women were grouped by age (<40, 40–50, >50 years), corresponding to pre-, peri- and post-menopausal stages. EIC, HIV VL suppression (VL < 50 copies/mL) within 12 months of antiretroviral therapy initiation and VL rebound (two consecutive VL > 50 copies/mL) after VL suppression were compared across age groups using logistic/Cox proportional hazards regression. Associations were compared to those seen in heterosexual men.

**Results:** Six thousand four hundred and fifty-five (6455) eligible women (median age 36 [interquartile range: 29–42], 64.4% black African, 19.1% white) contributed 44,226 person-years (PYRS) of follow-up; 29,846, 10,980 and 3,399 PYRS in those aged <40, 40–50 and >50, respectively. Women were engaged-in-care for 79.5% of follow-up time, 3,344 (78.0%) experienced VL suppression and 739 (22.1%) VL rebound. After adjustment, women aged >50 years had lower EIC than those aged <40. Women aged 40–50 were more likely to have VL suppression and were less likely to experience VL rebound than those aged <40 years. Trends in heterosexual men were similar for EIC but with no evidence of a higher VL suppression rate in those aged 40–50 years (*p*_int._ 0< .0001) and a stronger protective association between older age and VL rebound (*p*_int._ 0< .0001).

**Conclusion:** Our findings warrant further research into the potential impact of the menopause to support women and clinicians through HIV care.

## Introduction

In the UK, nearly a third of those living with HIV are women. With successes in treatment, the increasing life expectancy of people living with HIV now means this population is ageing; in 2018, 4.2% of women living with HIV were transitioning through the menopause and 33% were post-menopausal,^1^ with these proportions expected to increase in the coming decade.^2^

Women undergo sex-specific changes as they age, specifically with hormone regulation. There is limited evidence available with regards to how these changes, more specifically the menopause, impact HIV outcomes. It is therefore imperative to understand the impact of the menopause on HIV care.^3^

Only a few studies have specifically examined the association between menopausal symptoms and adherence to antiretroviral therapy (ART).^4–7^ A recent analysis of the Positive Transitions through the Menopause (PRIME) study, a cross-sectional study of women living with HIV in the UK aged 45–60 years, reported a significant association between severe menopausal symptoms and both sub-optimal adherence and HIV clinic attendance.^7^ Despite the significant impact of sub-optimal adherence and poor clinic attendance on clinical outcomes,^8^^,^^9^ there are currently no clear data on whether clinical HIV outcomes change among women as they progress through different stages of the menopause.

We aim to investigate whether menopausal age is associated with engagement-in-care and virological outcomes (viral load suppression and rebound) among women participating in the UK Collaborative HIV Cohort (UK CHIC) study. Given that age and menopausal status are highly correlated, and that associations between menopause and outcomes may reflect ageing rather than menopause *per se*, we also examine trends in men.

## Methods

The UK CHIC study is an ongoing cohort of individuals living with HIV (aged >16 years) who have accessed care at one or more of 25 HIV clinics in the UK at any time from 1996 onwards. The study methods are described elsewhere.^10^ In brief, centers collect data on demographic information, ART treatment history, laboratory results and AIDS diagnoses; the resulting dataset is submitted on an annual basis to the coordinating center. The project was approved by a Multicentre Research Ethics Committee (MREC/00/7/47) and by local ethics committees.

The analyses described here are based on women who acquired HIV through heterosexual sex only, as factors associated with HIV outcomes may differ in those acquiring HIV through other routes, and as the number of women acquiring HIV through these routes in the cohort is too small to permit robust estimates. Data were collected up to 31 December 2017. Heterosexual women with >1 day of follow-up and a cohort entry date in January 2000 or later were followed from cohort entry until the earliest of the following: death, permanent loss to follow-up from HIV care (defined as failure to return for a follow-up visit within 12 months) or 31st December 2017. As UK CHIC does not record menopause specific data, women were grouped by age (<40, 40–50, >50 years) to broadly corresponding to pre-, peri- and post-menopausal stages.

Each woman’s follow-up was split into consecutive monthly intervals and the woman’s characteristics (age, CD4+ T-cell count, HIV viral load (VL), combination ART (cART) use, previous AIDS diagnosis and calendar year) were determined at the start of each interval, allowing us to incorporate time-varying covariates into regression models. Ethnicity and Hepatitis B virus (HBV)/Hepatitis C virus (HCV) co-infection were treated as fixed covariates.

Analyses considered three outcomes: (i) Engagement-in-care (EIC), which was defined using the Retention and Engagement Across Care services for HIV (REACH) algorithm^11^ in which a person’s clinical status is used to estimate the likely time to the next scheduled follow-up appointment – based on this information, each person-month is classified as being “in-care” or “out-of-care” according to whether the person had a return visit within the expected time interval; (ii) VL suppression, defined as an initial VL ≤50 copies/mL in the subset of women who initiated cART and (iii) VL rebound, defined as two consecutive VL >50 copies/mL among women previously virologically suppressed on cART.

Univariable and multivariable regression were used to assess the association between age group and each outcome, adjusting for potential confounders selected *a priori*. These included: ethnicity, CD4+ T-cell count, VL, ART use, previous AIDS, calendar year and hepatitis B/C infection. Analyses of EIC used generalized estimating equations (GEE) to model the association between age and the binary outcome of whether each month of follow-up was deemed to be in or out of care. For the analyses of viral suppression, individuals who initiated cART were followed from cART initiation to the earliest of the censoring date (described above) or 12 months after cART initiation. Time to VL suppression was compared across the three age groups using Cox proportional hazards regression. Among those with VL suppression, time to viral rebound was assessed from the date of VL suppression to the earliest of VL rebound, the censoring date or the first gap in treatment lasting >14 days, with comparisons between the age groups undertaken using Cox proportional hazards regression models.

We undertook further analyses within the whole UK CHIC cohort (including both men and women), to assess whether age associations (where present) differed significantly between men and women, through the inclusion of an interaction term between age group and sex.

## Results

A total of 6,455 heterosexual women in UK CHIC were included in analyses (Table 1); the women were enrolled at a median age of 36 [interquartile range, IQR: 29–42] years, and with a median CD4+ T-cell count of 287 [IQR: 140–463] cells/mm^3^. The majority of women were of black African ethnicity (70.6%). One-tenth (9.5%) of women had a prior AIDS event at study entry, and 0.4% and 0.2% were co-infected with HBV or HCV, respectively.

**Table 1:** Demographic and clinical characteristics at time of entry to UK CHIC of the heterosexual women included in the study, overall and stratified by age group at study entry

Variable		All women (*n* = 6455)	Age group at entry to UK CHIC study (years)		
			<40 (*n* = 4253)	40–50 (*n* = 1595)	>50 (*n* = 607)
Ethnicity	White	905 (14.0)	583 (13.7)	203 (12.7)	119 (19.6)
	Black Caribbean	366 (5.7)	201 (4.7)	99 (6.2)	66 (10.9)
	Black African	4560 (70.6)	3061 (72.0)	1140 (71.5)	359 (59.1)
	Black Other	233 (3.6)	146 (3.4)	63 (4.0)	24 (4.0)
	South Asian/Other Asian	173 (2.7)	112 (2.6)	42 (2.6)	19 (3.1)
	Mixed/Other	218 (3.4)	150 (3.5)	48 (3.0)	20 (3.3)
Year of study entry	2000–2006	2764 (42.8)	2099 (49.4)	529 (33.2)	136 (22.4)
	2007–2011	2469 (38.3)	1532 (36.0)	692 (43.4)	245 (40.4)
	2012–2017	1222 (18.9)	622 (14.6)	374 (23.5)	226 (37.2)
CD4+ T-cell count at study entry (cells/mm^3^)	<200	2003 (35.9)	1209 (33.1)	590 (42.1)	204 (39.2)
	200–349	1374 (24.7)	909 (24.9)	332 (23.7)	133 (25.5)
	350–500	1012 (18.1)	697 (19.1)	239 (17.1)	76 (14.6)
	>500	1185 (21.3)	837 (22.9)	240 (17.1)	108 (20.7)
AIDS at study entry		616 (9.5)	357 (8.4)	180 (11.3)	79 (13.0)
HBV at study entry		28 (0.4)	15 (0.4)	10 (0.6)	3 (0.5)
HCV at study entry		14 (0.2)	10 (0.2)	3 (0.2)	1 (0.2)

HBV, hepatitis B virus; HCV, hepatitis C virus.

The women were followed for a total of 44,226 person-years (PYRS); 29,846, 10,980 and 3,399 PYRS were contributed by women aged <40, 40–50 and >50 years, respectively. There were several key differences in the characteristics of the women in the three age groups. For example, women in the older age group tended to be followed up in more recent calendar years, generally had higher CD4+ T-cell counts, were more likely to have had a prior AIDS event, and were more likely to be on cART and to have a suppressed VL (see Table 2).

**Table 2:** Follow-up time (person-years) contributed by women included in the study with different demographic and clinical characteristics, overall and percentage stratified by age group

Variable		Total person-years follow-up	Age (years)		
			<40	40–50	>50
Calendar year	2000–2006	95,552	30.3	12.7	7.2
	2007–2011	163,156	37.2	32.3	23.4
	2012–2017	238,240	32.4	55.0	69.5
CD4+ T-cell count (cells/mm^3^)	<200	42,246	10.9	8.4	7.5
	200–349	83,811	21.8	16.9	14.2
	350–500	113,941	26.9	24.4	22.1
	>500	216,117	40.3	50.3	56.3
Virally suppressed	No	188,686	52.5	29.0	23.0
	Yes	308,262	47.5	71.0	77.0
Previous AIDS	No	388,809	82.5	75.8	73.5
	Yes	108,139	17.5	24.2	26.5
Ever initiated ART	No	145,145	40.5	22.2	17.8
	Yes	351,803	59.5	77.8	82.2

ART, antiretroviral therapy.

### Engagement-in-care

Women were determined to be engaged-in-care for 79.5% of their follow-up time, with the proportion of follow-up time spent engaged-in-care increasing from 73.6% among the younger age group to 82.0% in the middle age group to 85.9% in the older age group. Compared to those aged <40 years, in unadjusted analyses the odds of EIC increased by 27% (odds ratio (OR) 1.27 [95% confidence interval: 1.19–1.36]) and 41% (1.41 [1.27–1.57]) among those aged 40–50 and >50 years, respectively. After adjustment, however, both ORs fell below 1 (40–50 years: adjusted OR (aOR): 0.96 [0.89–1.03]; >50 years: 0.87 [0.78–0.96]) suggesting a gradual decrease in EIC as age increased.

### Viral load suppression

A total of 3,344 (78.0%) women who initiated cART became virally suppressed within the first 12 months (median time to VL suppression 4 months [IQR: 2–8 months]). Women initiating cART aged 40–50 years were more likely to become virally suppressed within a year (82.1%) than those aged <40 (75.8%) or >50 (78.9%) years, with crude hazard ratios (HRs) for those aged 40–50 and >50 years at cART initiation of 1.16 [1.08–1.25] and 1.06 [0.95–1.18], respectively, compared to those aged <40 years. After adjustment for potential confounders, the adjusted hazard ratio was increased for women aged 40-50 years (adjusted HR (aHR) 1.25 [1.14-1.37]) but remained similar for women aged >50 years (aHR: 1.08 [0.94–1.23]).

### Viral rebound

Over one-fifth (22.1%) of women with viral suppression subsequently experienced virological rebound over 12,307 PYRS. Cumulatively, 13.9%, 20.1% and 23.9% had a viral rebound within the first two, four and six years after viral suppression, respectively. In unadjusted analyses, whilst VL rebound rates among women aged <40 and 40–50 years did not differ significantly (HR: 0.90 [0.77–1.06] for women aged 40–50 years compared to those aged <40 years), women aged >50 years were less likely to experience viral rebound (HR: 0.70 [0.56–0.88]) than those aged <40 years. After adjustment for confounders, however, the aHR for women aged 40–50 years was strengthened (aHR: 0.82 [0.68–0.98]) whereas that for women aged >50 years was attenuated (aHR: 0.89 [0.69–1.15]).

### Comparison with men in UK CHIC

A total of 5,843 heterosexual men from the UK CHIC study were included in comparative analyses, consisting of 3199 men aged <40 years, 1835 aged 40–50 years and 809 aged >50 years at study entry. This group contributed a total follow-up of 41,620 PYRS. Compared to women, heterosexual men in UK CHIC were older at study entry (median age: 38 [IQR: 32–46] years), with a lower median CD4+ T-cell count (264 [IQR: 115–430] cells/mm^3^). A greater proportion were of white ethnicity (24.6%). As observed in women, the proportions with a prior AIDS event (11.1%), HBV and HCV co-infection (0.7% and 0.3%, respectively) were low.

**Figure 1:**
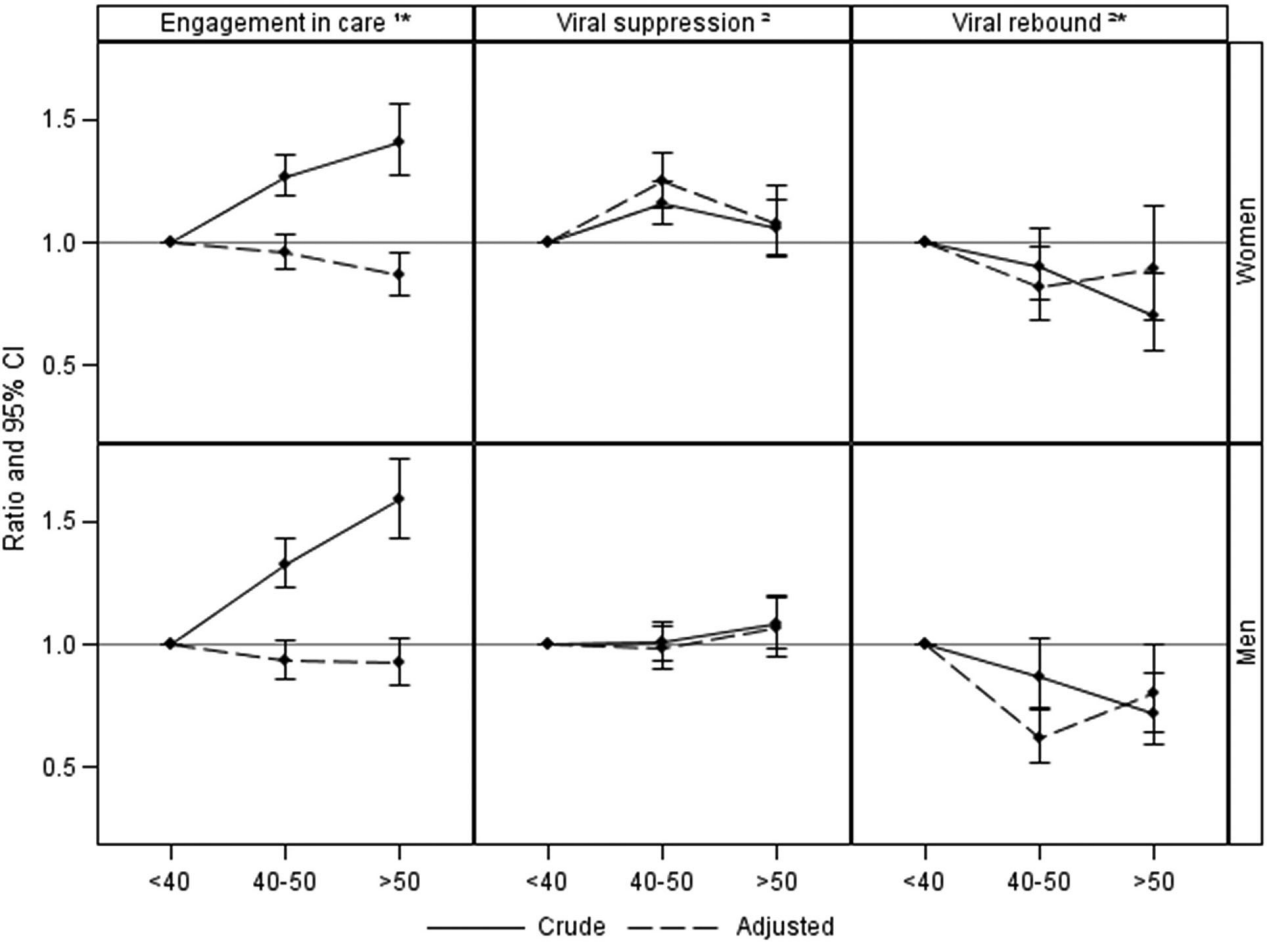
Crude and adjusted odds/hazard ratio of engagement in care*, time to viral suppression and viral rebound* at peri- and post-menopausal age compared to pre-menopausal age. Adjusted for ethnicity, ever started cART, previous AIDS, HBV/HCV, calendar year, CD4+ T-cell count, HIV viral load. CI, confidence intervals; ^1^logistic regression; ^2^Cox proportional hazard model; * time-updated covariates.

Among the men, rates of EIC also showed an increasing trend with age (72.5%, 82.0% and 85.9% in the three age groups, respectively) although after adjustment for potential confounders, there were no significant differences in rates of EIC across the age groups (age 40–50: aOR 0.94 [0.86–1.02]; age >50: 0.92 [0.83–1.03] compared to age <40 years; Figure 1). Although the association between EIC and men aged >50 appeared to be weaker than that seen in women aged >50, a test of interaction suggested that the association between age and EIC did not differ significantly between men and women (*p* = 0.36).

In total, 3,353 (80.1%) of the men became virally suppressed within the first 12 months on cART, 78.8%, 80.2% and 83.6% in the three age groups, respectively. In contrast to women, there was no association between viral suppression and age amongst men after adjustment for confounding (age 40–50: aHR 0.98 [0.90–1.08]; age >50: aHR 1.07 [0.95–1.20] vs. <40 years; Figure 1) and a test of interaction confirmed that the association between age and viral suppression differed by sex (*p* < 0.0001).

Viral rebound occurred in 750 (22.4%) of those with viral suppression (25.4%, 19.9% and 19.1% of the three groups, respectively). Associations with age appeared to be stronger amongst heterosexual men (age 40–50: aHR 0.62 [0.51–0.74]; age >50: aHR 0.80 [0.65–0.99]; Figure 1), with older ages appearing to be more strongly associated with a reduced risk of viral rebound than in women (*p* < 0.0001 for test of interaction).

## Discussion

Using data on women participating in a large cohort study of people with HIV in the UK, we have examined associations of EIC and virological outcomes with age groups broadly corresponding to pre-, peri- and post-menopausal age. Whilst there was no significant difference in EIC between women in the age groups broadly corresponding to pre- and peri-menopausal stages, we found a lower likelihood of engaging in care amongst women of a post-menopausal age. However, there were no differences between age and EIC in heterosexual men and women. Women of a peri-menopausal age were more likely to attain VL suppression and less likely to have a VL rebound compared to pre-menopausal aged women. This was in contrast to the pattern seen amongst the older age categories of heterosexual men, where there was no difference in the rate of viral suppression and a consistent decline in rate of viral rebound.

Our analysis suggests post-menopausal aged women may experience difficulties in engaging with care. The most recent PRIME study is the first in England to report on the associations between EIC and menopausal status. Duff et al. reported no differences in EIC by menopausal status, but severe menopausal symptoms were found to have a negative impact on EIC.^4^ In contrast to our study, previous analyses of the UK CHIC Study utilizing the REACH algorithm suggested a greater rate of EIC amongst individuals older than 45 years rather than a lower rate.^12^ Our results also differ from other studies that have explored the association between age and EIC/clinic nonattendance.^13^^,^^14^ Both Olatosi et al. and Kiplagat et al. reported that people living with HIV aged ≥55 and ≥50, respectively, were more likely to be engaged in care compared to their younger counterparts. However, neither study reported results after disaggregation by sex.

It is important, however, to note differences in definitions of EIC used in the studies, with Olatosi et al. defining EIC as at least one measured CD4+ T-cell count or viral load test within each calendar year,^13^ and Kiplagat et al. defining this as no clinical attendance within three months after the last clinical visit date (among those not recorded as dead or transferred out of care).^14^ We used the REACH algorithm, an agile tool allowing us to predict expected date of next clinic attendance according to guidelines, and applied it to routinely collected clinical data over a prolonged period of time. The flexibility of the algorithm may result in a more sensitive measure of EIC.^11^^,^^15^

In our study, we found that peri-menopausal aged women (those aged 40–50 years) were more likely be virally suppressed and less likely to experience viral rebound than women aged <40. There have been only two studies to our knowledge that have explored the association of virological suppression with reported menopausal status.^6^^,^^16^ In contrast to our findings, both studies reported no difference in viral suppression rates according to menopausal status amongst women in Brazil and Spain. The data on age and viral suppression are conflicting, with some studies suggesting that older people living with HIV have higher rates of VL suppression^17–21^ and others reporting no association with age.^22–25^ Studies conducted in Latin America/the Caribbean and South Africa have similarly suggested a lower likelihood of experiencing VL rebound amongst older men and women living with HIV.^25^^,^^26^ Our results may support a greater level of adherence to ART in heterosexual women of a peri-menopausal age in this setting, although given previous published data, our findings should be interpreted cautiously. Although there are currently limited data exploring HIV outcomes amongst older people with HIV in a gender-disaggregated manner, older individuals have generally been reported to have better adherence and tolerability to ART.^11^ Further research exploring this is required to tease out possible explanations between onset of menopause and ART adherence or tolerability.

We report no differences in the association between age and EIC in heterosexual men and women of similar age, suggesting that any reduction in EIC in the older age group may be related to ageing, rather than menopause specifically. In contrast, when exploring the association between age and viral outcomes, we found that the association differed significantly between men and women. Women of peri-menopausal age (40–50 years) had a higher likelihood of VL suppression compared to women aged <40 years, but this association was not present in men. Additionally, women in the peri-menopausal age group were less likely to experience virological failure compared to women aged <40, and this trend was not observed in the post-menopausal group. However, older men were consistently less likely to have VL rebound in both older age groups (40–50 and >50) compared to men aged <40 years. Previous analyses of the UK CHIC Study and American datasets exploring virological responses have reported no differences by sex.^27^^,^^28^ Although, a Canadian study found that women were more likely to attain VL suppression and viral rebound than men,^29^ these associations were not explored in relation to age specifically. Our study uniquely suggests that women of a peri-menopausal age may undergo changes, which directly or indirectly impact adherence or reduced toxicity to ART. For example, we could speculate that the onset of menopausal symptoms in the peri-menopausal group may cause concern among women prompting attendance at the HIV clinic with associated adherence support, therefore resulting in higher rates of VL suppression and lower rates of viral rebound at peri-menopausal ages.

A strength of our study is the large number of women living with HIV from a representative sample of people attending HIV care in the UK included in our analyses. Longitudinal follow-up through routinely collected data in the UK CHIC Study cohort allows us to understand dynamic changes in clinical outcomes as people with HIV age.

A key limitation is that we have no data on menopause status or menopausal symptoms, and instead use age as a surrogate marker. Although we selected our age groups to broadly correspond to menopausal stage, it is important to acknowledge that they may not capture this perfectly. Our age groups for this analysis were selected on the basis of the age distribution of women with HIV in the UK CHIC cohort as well as the expected age at onset of menopausal symptoms (with an average age of menopause in the UK of 51 years).^30^ Although further stratification of age into finer age groups may have provided a more accurate separation of those with and without menopausal symptoms, the number of women over the age of 50 in the cohort was too small to permit reliable and appropriately powered analyses. Sensitivity analyses (not shown) suggested that associations did not differ substantially when alternative age groupings were used.

Additionally, although our analyses focus on menopause, we recognize that many other factors may contribute to the differences seen in the age groups selected. Younger women, for example, may experience barriers which negatively impact their ability to engage in healthcare and to adhere to therapy, particularly when caring for a family. Ageing in general represents a whole host of new challenges not considered in these analyses, e.g. diagnoses of new comorbidities that may lead to polypharmacy. As such, the possibility of drug-drug interactions must always be considered to ensure there is no loss in virological efficacy or increased drug toxicities as people age.^31^^,^^32^ Furthermore, it is important to acknowledge the psychosocial impact of factors such as caring responsibilities and declines in health over a woman’s lifetime, with consequent effects on mental health.^33^^,^^34^

Further limitations include a lack of lifestyle data (e.g. alcohol consumption, smoking habits) which may have an impact on the timing and symptoms of menopause as well as ART adherence.^35^ The majority of follow-up time in the present study occurred from 2012 to 2017, a period of time during which individuals would have had access to more tolerable ART drugs, therefore the risk of adverse events are rare. This may potentially reduce any negative impact of menopausal status on adherence. And lastly, our cohort is representative of the UK’s heterosexual HIV epidemic and results cannot be generalized to other populations in the UK and beyond.

In summary, using data from one of the largest and representative cohorts of people with HIV, we find an association between peri-menopausal age and viral outcomes among heterosexual women living with HIV. We suggest there is better adherence to ART amongst women aged 40–50 compared to that in younger women. Our findings highlight the importance of taking a life course approach when providing care to women living with HIV, and ensuring support is tailored to their needs at different life stages. We, therefore, welcome the national recommendations that HIV clinics provide information about the menopause to women attending their service, and establish care pathways with specialist menopause services and primary care.^36^ Further research to understand the relationship between the onset of menopause and HIV outcomes is imperative to inform both women living with HIV and clinicians responsible for their care of the impact of the menopause on their health and well-being.

## References

[1] Brown A, Rawson S, Kelly C, et al. *Women and HIV in the UK: October 2019*. London: Public Health England; 2019.

[2] Mahy M, Autenrieth CS, Stanecki K, et al. Increasing trends in HIV prevalence among people aged 50 years and older. *AIDS* 2014;28:S453–S459. 25222641 10.1097/QAD.0000000000000479 PMC4247270

[3] Solomon D, Sabin CA, Mallon PWG, et al. Cardiovascular disease in women living with HIV: a narrative review. *Maturitas* 2018;108:58–70. 29290216 10.1016/j.maturitas.2017.11.012

[4] Duff PK, Money DM, Ogilvie GS, et al. Severe menopausal symptoms associated with reduced adherence to antiretroviral therapy among perimenopausal and menopausal women living with HIV in Metro Vancouver. *Menopause* 2018;25:531–537. 29206769 10.1097/GME.0000000000001040 PMC5899045

[5] Cutimanco-Pacheco V, Arriola-Montenegro J, Mezones-Holguin E, et al. Menopausal symptoms are associated with non-adherence to highly active antiretroviral therapy in human immunodeficiency virus-infected middle-aged women. *Climacteric* 2020;23(3):229–228. 31809600 10.1080/13697137.2019.1664457

[6] Calvet GA, Velasque L, Luz PM, et al. Absence of effect of menopause status at initiation of first-line antiretroviral therapy on immunologic or virologic responses: a cohort study from Rio de Janeiro, Brazil. *PLoS One* 2014;9(2):e89299. 24586673 10.1371/journal.pone.0089299 PMC3930701

[7] Solomon D, Sabin CA, Burns F, et al. The association between severe menopausal symptoms and engagement with HIV care and treatment in women living with HIV. *AIDS Care* 2020:1–8. 10.1080/09540121.2020.1748559 PMC8043570 32279528

[8] Apisarnthanarak A, Mundy LM. Long-term outcomes of HIV-infected patients with <95% rates of adherence to nonnucleoside reverse-transcriptase inhibitors. *Clin Infect Dis.* 2010;51:115–117. 10.1086/653445 20518676

[9] Bastard M, Pinoges L, Balkan S, et al. Timeliness of clinic attendance is a good predictor of virological response and resistance to antiretroviral drugs in HIV-infected patients. *PloS One* 2012;7(11):e49091. 23145079 10.1371/journal.pone.0049091 PMC3492309

[10] Committee UCHCS. The creation of a large UK-based multicentre cohort of HIV-infected individuals: The UK Collaborative HIV Cohort (UK CHIC) Study. *HIV Med.* 2004;5:115–124. 15012652 10.1111/j.1468-1293.2004.00197.x

[11] Howarth AR, Burns FM, Apea V, et al. Development and application of a new measure of engagement in out-patient HIV care. *HIV Med.* 2017;18(4):267–274. 27535219 10.1111/hiv.12427 PMC5347876

[12] Howarth A, Apea V, Michie S, et al. REACH: a mixed-methods study to investigate the measurement, prediction and improvement of retention and engagement in outpatient HIV care. *Health Serv Deliv Res.* 2017;5(13):1–160. 28368560

[13] Olatosi BA, Probst JC, Stoskopf CH, et al. Patterns of engagement in care by HIV-infected adults: South Carolina, 2004-2006. *AIDS* 2009;23(6):725–730. 19197194 10.1097/QAD.0b013e328326f546

[14] Kiplagat J, Mwangi A, Keter A, et al. Retention in care among older adults living with HIV in western Kenya: a retrospective observational cohort study. *Plos One* 2018;13(3):e0194047. 29590150 10.1371/journal.pone.0194047 PMC5874021

[15] Sabin CA, Howarth A, Jose S, et al. Association between engagement in-care and mortality in HIV-positive persons. *AIDS* 2017;31(5):653–660. 28060018 10.1097/QAD.0000000000001373 PMC5333728

[16] Alejos B, Suarez I, Sanz N, et al., Does menopause affect treatment response in HIV-infected women? A multicenter cohort in Spain. InL 17th European AIDS Conference, Basel; 2019.

[17] Greenbaum AH, Wilson LE, Keruly JC, et al. Effect of age and HAART regimen on clinical response in an urban cohort of HIV-infected individuals. *AIDS* 2008;22:2331–2339. 18981772 10.1097/QAD.0b013e32831883f9 PMC2597671

[18] COHERE Group. Response to combination antiretroviral therapy: variation by age. *AIDS* 2008;22:1463–1473. 18614870 10.1097/QAD.0b013e3282f88d02

[19] Ghidei L, Simone MJ, Salow MJ, et al. Aging, antiretrovirals, and adherence: a meta analysis of adherence among older HIV-infected individuals. *Drugs Aging* 2013;30(10):809–819. 23959913 10.1007/s40266-013-0107-7 PMC3844933

[20] Kowalska JD, Kubicka J, Siwak E, et al. Factors associated with the first antiretroviral therapy modification in older HIV-1 positive patients. *AIDS Res Ther.* 2016;13(1):2. 26744599 10.1186/s12981-015-0084-5 PMC4704295

[21] Zhang Q, Yu X, Wu T, et al. Immunological and virological responses in older HIV-infected adults receiving antiretroviral therapy: an evidence-based meta-analysis. *J Acquir Immune Defic Syndr.* 2020;83(4):323–333. 31913990 10.1097/QAI.0000000000002266

[22] Orlando G, Meraviglia P, Cordier L, et al. Antiretroviral treatment and age-related comorbidities in a cohort of older HIV-infected patients. *HIV Med.* 2006;7(8):549–557. 17105515 10.1111/j.1468-1293.2006.00420.x

[23] Althoff KN, Justice AC, Gange SJ, et al. Virologic and immunologic response to HAART, by age and regimen class. *AIDS* 2010;24:2469–2479. 20829678 10.1097/QAD.0b013e32833e6d14 PMC3136814

[24] Szadkowski L, Tseng A, Walmsley SL, et al. Short communication: effects of age on virologic suppression and CD4 cell response in HIV-positive patients initiating combination antiretroviral therapy. *AIDS Res Hum Retroviruses.* 2012;28(12):1579–1583. 22734840 10.1089/AID.2012.0018

[25] Dawood H, Hassan-Moosa R, Zuma N-Y, et al. Mortality and treatment response amongst HIV-infected patients 50 years and older accessing antiretroviral services in South Africa. *BMC Infect Dis.* 2018;18(1):168. 29636023 10.1186/s12879-018-3083-z PMC5894176

[26] Carriquiry G, Giganti MJ, Castilho JL, et al. Virologic failure and mortality in older ART initiators in a multisite Latin American and Caribbean Cohort. *J Intern AIDS Soc.* 2018;21(3):e25088. 10.1002/jia2.25088 PMC5864576 29569354

[27] Barber TJ, Geretti AM, Anderson J, et al. Outcomes in the first year after initiation of first-line HAART among heterosexual men and women in the UK CHIC Study. *Antivir Ther.* 2011;16(6):805–814. 21900712 10.3851/IMP1818

[28] Patterson K, Napravnik S, Eron J, et al. Effects of age and sex on immunological and virological responses to initial highly active antiretroviral therapy. *HIV Med.* 2007;8(6):406–410. 17661850 10.1111/j.1468-1293.2007.00485.x

[29] Raboud J, Blitz S, Walmsley S, et al. Effect of gender and calendar year on time to and duration of virologic suppression among antiretroviral-naïve HIV-infected individuals initiating combination antiretroviral therapy. *HIV Clin Trials.* 2010;11(6):340–350. 21239362 10.1310/hct1106-340

[30] The National Institute of Health and Care Excellence (NICE) Guideline. Menopause: Diagnosis and Management. 33141539

[31] Tseng A, Szadkowski L, Walmsley S, et al. Association of age with polypharmacy and risk of drug interactions with antiretroviral medications in HIV-positive patients. *Ann Pharmacother.* 2013;47(11):1429–1439. 24285760 10.1177/1060028013504075

[32] Edelman EJ, Gordon KS, Glover J, et al. The next therapeutic challenge in HIV: polypharmacy. *Drugs Aging.* 2013;30(8):613–628. 23740523 10.1007/s40266-013-0093-9 PMC3715685

[33] Sherr L, Molloy A, Macedo A, et al. Ageing and menopause considerations for women with HIV in the UK. *J Virus Erad.* 2016;2(4):215–218. 27781103 10.1016/S2055-6640(20)30874-8 PMC5075348

[34] Loutfy M, Andany N, Kennedy VL, et al. Perspectives on menopause and women with HIV. *Int J Women's Health.* 2016;8:1–22. 26834498 10.2147/IJWH.S62615 PMC4716718

[35] Imai K, Sutton MY, Mdodo R, et al. HIV and menopause: a systematic review of the effects of HIV infection on age at menopause and the effects of menopause on response to antiretroviral therapy. *Obstet Gynecol Int.* 2013;2013:1–11. 10.1155/2013/340309 PMC3880754 24454386

[CIT0036] 36.British HIV Association. *2018 Standards of Care for People Living with HIV*. London: BHIVA; 2018.

